# The Intersection of the Extrinsic Hedgehog and WNT/Wingless Signals with the Intrinsic Hox Code Underpins Branching Pattern and Tube Shape Diversity in the *Drosophila* Airways

**DOI:** 10.1371/journal.pgen.1004929

**Published:** 2015-01-23

**Authors:** Ryo Matsuda, Chie Hosono, Kaoru Saigo, Christos Samakovlis

**Affiliations:** 1 Department of Molecular Biosciences, The Wenner-Gren Institute, Stockholm University, Stockholm, Sweden; 2 Department of Biophysics and Biochemistry, Graduate School of Science, University of Tokyo, Tokyo, Japan; 3 ECCPS, University of Giessen, Giessen, Germany; Harvard Medical School, Howard Hughes Medical Institute, UNITED STATES

## Abstract

The tubular networks of the *Drosophila* respiratory system and our vasculature show distinct branching patterns and tube shapes in different body regions. These local variations are crucial for organ function and organismal fitness. Organotypic patterns and tube geometries in branched networks are typically controlled by variations of extrinsic signaling but the impact of intrinsic factors on branch patterns and shapes is not well explored. Here, we show that the intersection of extrinsic *hedgehog*(*hh*) and *WNT/wingless* (*wg*) signaling with the tube-intrinsic Hox code of distinct segments specifies the tube pattern and shape of the *Drosophila* airways. In the cephalic part of the airways, *hh* signaling induces expression of the transcription factor (TF) *knirps* (*kni*) in the anterior dorsal trunk (DTa1). *kni* represses the expression of another TF *spalt major* (*salm*), making DTa1 a narrow and long tube. In DTa branches of more posterior metameres, Bithorax Complex (BX-C) Hox genes autonomously divert *hh* signaling from inducing *kni*, thereby allowing DTa branches to develop as *salm*-dependent thick and short tubes. Moreover, the differential expression of BX-C genes is partly responsible for the anterior-to-posterior gradual increase of the DT tube diameter through regulating the expression level of Salm, a transcriptional target of *WNT/wg* signaling. Thus, our results highlight how tube intrinsic differential competence can diversify tube morphology without changing availabilities of extrinsic factors.

## Author Summary

Tubes are common structural elements of many internal organs, facilitating fluid flow and material exchange. To meet the local needs of diverse tissues, the branching patterns and tube shapes vary regionally. Diametric tapering and specialized branch targeting to the brain represent two common examples of variations with organismal benefits in the *Drosophila* airways and our vascular system. Several extrinsic signals instruct tube diversifications but the impact of intrinsic factors remains underexplored. Here, we show that the local, tube-intrinsic Hox code instructs the pattern and shape of the dorsal trunk (DT), the main *Drosophila* airway. In the cephalic part (DT1), where Bithorax Complex (BX-C) Hox genes are not expressed, the extrinsic Hedgehog signal is epistatic to WNT/Wingless signals. Hedgehog instructs anterior DT1 cells to take a long and narrow tube fate targeting the brain. In more posterior metameres, BX-C genes make the extrinsic WNT/Wingless signals epistatic over Hedgehog. There, WNT/Wingless instruct all DT cells to take the thick and short tube fate. Moreover, BX-C genes modulate the outputs of WNT/wingless signaling, making the DT tubes thicker in more posterior metameres. We provide a model for how intrinsic factors modify extrinsic signaling to control regional tube morphologies in a network.

## Introduction

Branched tubular networks, like our vasculature transport and exchange vital gases and nutrients along entire organisms. The branching patterns, tube structures and dimensions in these networks show considerable regional variations to meet the different needs of target organs and ensure optimal organ function and animal fitness [1–4]. Adaptations of branch morphologies to the tissue environments can be achieved by changing the local availability of extrinsic factors like guidance molecules and/or by intrinsic regional differences in tube cell competence to respond and modify signaling outcomes. Although the prominent roles of variations in extrinsic signals in organotypic branching become widely established [3,5,6], the tube intrinsic mechanisms determining the differential responses of tube cells to signaling remain to be explored [7–10].

Despite the huge evolutionary distance of insects and mammals, the formation and maturation of the respiratory tube network in *Drosophila melanogaster* has served as a fruitful model system of branching morphogenesis [11,12]. Here, we use this system to evaluate the contribution of tube intrinsic, regionally differential competence in diversification of tube morphology.

**Figure 1:**
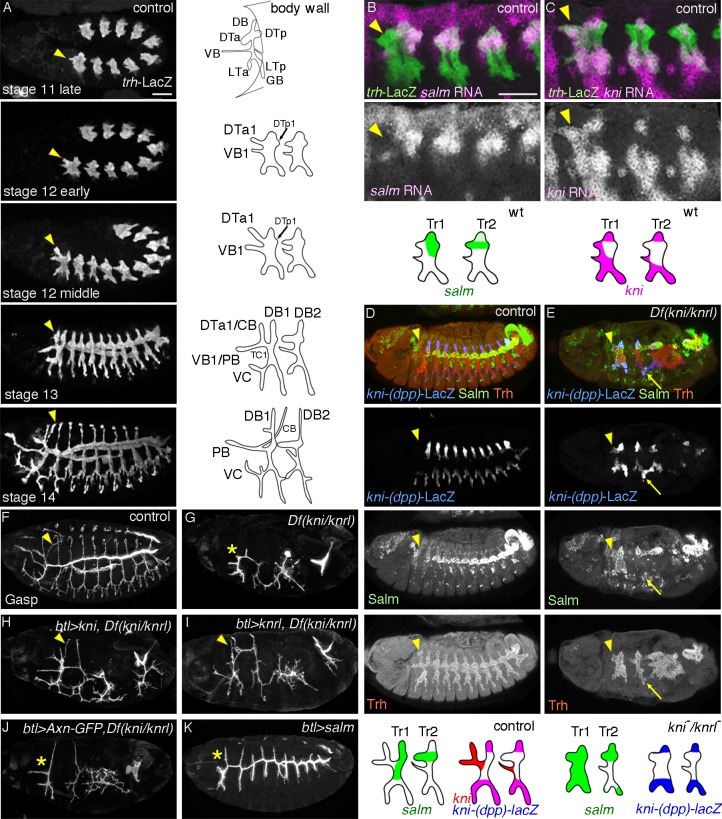
Developmental and genetic distinction between DTa1 and the rest of DT. Embryos are aligned with their anterior left and dorsal up unless otherwise indicated. (A), Times series of airway development visualized with *trh-lacZ*. DTa1 is marked with arrowheads. Schemes of the typical primary branches or the metamere 1/2 are shown on the right. See text for details. (B, C), Late stage11 embryos marked with *trh*-LacZ and RNA *in situ* hybridization against *salm* (B) or *kni* (C). Typically, DT and DB initiate *salm* expression while *kni* expression represses *salm* later in the DB. Note that DTa1 (arrowheads) expresses *kni* but not *salm*.(D, E) Stage 13 control and *Df(kni/knrl)* embryos marked with *kni-(dpp)*-LacZ, Salm and Trh. Schemes of their expression are shown in the bottom. In the control (D), Salm is detected in DTp1-DT10. Salm is also reproducibly detected in DB1/10 and TC1. *kni-(dpp)*-LacZ marks subsets of Kni positive branches (DB/TC/GB) responding to *dpp* signaling. *kni-(dpp)*-LacZ and Salm overlap only in DB1/10. In *Df(kni/knrl)* (E), some abdominal segments are missing or fused. Note that *kni-(dpp)*-LacZ positive cells co-express Salm in DB, LT or GB (arrow) and that the whole terminal metameres, presumably Tr1/10, including DTa1 (arrowhead) express Salm. (F-K) Airway branching revealed by Gasp expression at stage 15. DTa1/CB is marked with arrowheads or asterisks. Compared to the control (F), in *Df(kni/knrl)* (G), *kni* positive branches (DB, LT, GB, VB and DTa1) are stalled or missing. Branches in Tr1 and Tr10 become very thick. *btl-gal4* driven *UAS-kni* (H) or *UAS-knrl* (I) significantly recues DTa1/CB formation. *btl-gal4* driven *UAS-Axn-GFP* (J) partially rescues DB extension without effects on dorsalward extension of CB while *btl-gal4* driven *UAS-salm* abrogates CB formation (K). Scales bars: 50um.

The fly respiratory network, also called the tracheal system ramifies extensively to deliver oxygen to each cell in the body (Fig. 1A) [13,14]. It derives from 10 primordial cell clusters specified in ectodermal para-segments (PS) 4–13 on each side of the body [15]. At stage 11, the metameric cell clusters invaginate and begin to extend 6 primary branches. The tracheal metameres (Tr1-10) interconnect into a network through the activities of 5 to 6 specialized fusion cells in each branching unit [16–18]. These cells find and adhere to their ipsilateral or contralateral counterparts in neighboring metameres to form continuous tubes.

Tracheal branching morphogenesis is controlled by two conceptually different groups of extrinsic signals [6,19]. One is required in all primary branches while the other class specifies unique sets of primary branches. FGF/Branchless (Bnl) [20] is dynamically expressed in the surrounding tissues and serves as a general extrinsic branching signal by activating FGFR/Breathless (Btl) on the airway cells [21–23]. Btl activation initiates oriented cell migration and enhances branch elongation while it also organizes cell fate specification along each primary branch. The gradual specification of distinct fusion and terminal cell fates at the branch tips is essential for both branch fusion and further ramification branching [14,20].

The second class of signals includes BMP, Wnts and Hh proteins. BMP/Decapentaplegic (Dpp) is expressed in dorsal and lateral stripes along the length of the embryo [24]. Dpp signaling specifies and licenses dorsally (dorsal branch, DB) and ventrally migrating primary branches (lateral trunk, LT and ganglionic branch, GB) by inducing the expression of the TFs *kni* and *knirps-related* (*knrl*) in airway cells [24–26]. Wg is expressed in a repetitive pattern of transverse ectodermal stripes and together with other WNT signaling molecules specifies dorsal trunk (DT) identity [27–29]. It upregulates the expression of the TF *salm* [30] in the major airways of the network. *salm* promotes short and thick tubes by suppressing intercalation of the tube constituent cells [31,32]. On the other hand, *kni/knrl* can repress *salm* expression in DB [25] and promotes cell intercalation in the long and narrow tubes of dorsal (DB) and ventral branches [31]. Hh is also segmentally expressed in ectodermal stripes and modulates the airway branching, both indirectly through positive regulation of *bnl* expression [33] and directly by acting on terminal cell specification or extension [34,35]. The functions of *hh* in primary branching remain to be thoroughly studied [34,36,37]. Collectively, the differential primary branch identities established by the second class of extrinsic signals are intimately linked with the distinctive branching patterns and dimensions of individual primary branches [38].

The region-specific modification of serially homologous organs and appendices is a general theme in animal development [39–42]. The evolutionary conserved Hox gene complexes are key selector genes of tissue identities along the anterior posterior (A-P) axis of animals [39,41,43–45] [46–50]. In *Drosophila*, 2 groups of Hox genes, the Antennapedia complex (ANTP-C) and Bithorax complex (BX-C) confer regional differences to the body plan by graded expression of their products in register with para-segmental units [39,51]. The 3 protein-coding genes of the BX-C [52] are expressed in distinct and partially overlapping domains along the A-P embryonic axis. *Ultrabithorax* (*Ubx*) expression initiates in the cells of PS5, *abdominal-A* (*abdA*) expression starts from PS7 and *Abdominal-B* (*AbdB*) from PS10 [44,53]. A connection between regional modification of airway morphology and Hox genes has been established already in the early studies of Hox-gene mutants. Upon loss of all BX-C genes the tracheal metameres Tr2-Tr10 become transformed to Tr1 [39,54]. However, the genetic and molecular mechanisms establishing the different branch morphologies along the airway tubes have been largely unexplored.

Here, we focus on the regulation of 2 distinct morphological modifications along the DT major airways. We first analyze how the most anterior part of the DT diverges its branching pattern and tube size to generate long and narrow tubes targeting the head. We show that these regional modifications in cell behaviors are controlled by a combination of *hh* signaling and the airway intrinsic Hox code. We further investigate the mechanism of tube tapering in the central domain of the DT airways. We find that BX-C genes modulate the anterior-to-posterior gradation of DT tube diameter partly through regulating the expression of level of *salm*, a target of *WNT*/*wg* signaling. Our work highlights that the intrinsic Hox code locally modifies the outcomes of extrinsic signals to establish regionally different branching patterns and to coordinate tube shapes in register with the embryo axis.

## Results and Discussion

### Specialized branching patterns and tube shapes in Tr1

The DT is a continuous tube running along the A-P axis of the embryo. It connects with the exterior through the narrow tube of the spiracular chamber in the posterior spiracle (PSP) [55]. The DT is constructed by the fusion of an anterior (DTa) and a posterior (DTp) branch from each tracheal metamere (Tr1-Tr10) [13,17]. The DT airway encompasses several regional variations that provide a suitable system for the study of the interplay of external signaling with intrinsic factors during tube morphogenesis [13]. First, the most anterior end of the DT extends several specialized branches (see below). Second, it shows a pronounced posterior to anterior diameter tapering contrasting the largely cylindrical shape of other primary branches in the network. Third, its most anterior metameric unit (Tr1) lacks a DTa fusion cell, whereas the most posterior one (Tr10) does not generate a fusion cell in its posterior branch (DTp).

More generally, Tr1 is distinct from the rest of the tracheal metameres because it encompasses more cells and branches to oxygenate the specialized organs of the head and thorax. The specialized branches of Tr1 include the cerebral branch (CB) targeting the brain, the pharyngeal branch (PB) to the anterior intestine, the ventral cephalic branch (VC) extending to epidermis and muscles and the ganglionic branch GB0, which penetrates the ventral nerve cord. Among these, the CB and VC are directly linked to the anterior end of the DT airways.

Despite the pronounced differences in the final branching patterns, branching in Tr1 is comparable to the common stereotypic primary branching of Tr2-Tr9 during stage 11 (Fig. 1A). At stage 12 however, DTa1, elongates further than other DTa branches and shifts dorsally. By stage 13, visceral branch 1 (VB1) and DTa1 co-segregate from the transverse connectives (TC). Later, DTa1 extends dorsally and posteriorly and turns towards the brain, forming CB [13]. DTa1/CB develops very narrow and long tubes compared to the thick and short DT branches in posterior metameres. VB1 extends more anteriorly, forming the pharyngeal branch (PB) (Fig. 1A).

### *kni* expression in DTa1/CB is essential for CB formation

The gradual morphological diversification of CB/DTa1 compared to DTa braches in other metameres prompted us to examine the expression of branch identity TFs, *salm* and *kni*. In a “typical”, central metamere, *salm* expression is upregulated by *wg/WNT* signaling [27–29] and is detected in DB and DT at stage 11 [30]. Later, *kni* is induced in DBs by *dpp*, where it represses *salm* expression [24–26]. *kni and salm* are co-expressed in DB1/10 (see below). In contrast to the DTa of other metameres, we found that *kni* expression is strongly upregulated in DTa1 from late stage 11 (Fig. 1B). Concomitantly, *salm* is not detectable in DTa1 (Fig. 1C) although it becomes upregulated in DTp1 as in other metameres (Fig. 1D) [30].

To test the significance of the differential *kni* expression in DTa1 we analyzed mutants lacking both *kni* and its paralog *knrl*. In these chromosomal deficiency mutants, the abdominal segments are missing due to the early gap gene function of *kni*, while trunk development is rather normal [56]. In the trunk region of *Df(kni/knrl)* mutants, the formation of *kni* positive primary branches [24,25] is variably affected, ranging from complete absence to branch stalling [25], while the *salm*-positive DT branches can form and fuse (see below) [57]. We noticed that in *kni/knrl* mutants, airway cells initiate branch outgrowth in the dorsal and ventral directions and respond to *dpp* by inducing the *dpp*-responsive kni reporter, *kni-(dpp)-lacZ* (Fig. 1E). In these embryos, *salm* is ectopically detected in *kni-(dpp)-lacZ* positive cells in either dorsal or ventral cells near the *wg* stripe (Fig. 1E). This suggests that *dpp* mediated *kni/knrl* induction suppresses *salm* induction by *wg* in both the dorsal [25,26] and ventral branches. However, in the first and the last metameres of *Df(kni/knrl)* mutants, *salm* expression additionally expands to nearly cover the entire metamere, including the putative CB/DTa1 (Fig. 1E). This suggests that *kni* functions in DTa1 to repress *salm*. Additionally, the competence of tracheal cells to induce *salm* is differentially modulated in the terminal Tr1 and Tr10 metameres compared to the central metameres.

Consistent with the notion that generalized reduction of *wg/WNT* signaling can bypass the requirement of *dpp/BMP* signaling during DB extension in Tr2-Tr10 [31], *btl*-Gal4 [58] mediated overexpression of GFP fused to Axin (Axn) [59–61], a negative regulator of *wg* signaling moderately rescues dorsal extension of the residual DBs (DB1 and DB2) of *Df(kni/knrl)* mutants but does not appreciably rescue the extension of DTa1/CB (Fig. 1J). In sharp contrast, *btl-gal4* driven *UAS-kni* or *UAS-knrl* in *Df(kni/knrl)* mutants restores CB formation (Fig. 1F-I). Thus, we conclude that *kni* induction and the resultant *salm* repression in DTa1/CB are essential for its formation and extension. In support for this, *btl-gal4* driven *UAS-salm* in wild-type background significantly suppresses DTa1/CB formation but has little effect on the extension of DTa in Tr2-Tr10 [28,62], which endogenously expresses *salm* (Fig. 1K). Collectively, the results suggest that *kni* induction and the resultant *salm* repression in DTa1/CB are essential for its formation and extension.

### *hh* is necessary for DTa1 patterning

The diversified expression of *kni* in DTa1 compared to the remaining DTa branches could be regulated by differential expression of exogenous guidance factors around DTa1 and/or by intrinsic differences of competence among the DT1 cells. Firstly, to investigate which extrinsic factors are upstream of *kni* induction in DTa1, we analyzed the expression or function of known, secreted airway branching regulators.

At stage 11, Tr1 like the rest of the tracheal metameres is surrounded by 6 patches of *bnl* expressing cells, prefiguring the stereotypic directions of the common primary branches (S1A Fig.). This general pattern diversifies in the cephalic region of stage 12 embryos, where the DTa1 migrates toward a more dorsal *bnl* expression spot (S1B Fig.). Despite this difference, *kni* induction in DTa1 is still detected in *btl* mutants (S1C,D Fig.), excluding a major role of *bnl* in *kni* induction in DTa1. *dEGFR/faint little ball/torpedo (top)* [63] encodes an RTK that is suggested to positively act on *salm* expression [64] upon binding the Spitz/EGF ligand [65]. In embryos mutant for *rhomboid (rho)*, encoding a protease required for generating the active Spitz *[66,67]* or for *dEGFR*, the expression of *kni* in the DTa1/CB still occurs (S1E,F Fig.). This argues against a role of localized dEGFR activation in controling *kni* expression in DTa1.

*dpp* and *wg* are known inducers of *kni/knrl* in DB [24] and *salm* in DT [27,28], respectively, in a “typical” metamere. Thus, variations of their expression in the Tr1 proximity might influence the specialized expression patterns of *kni* or *salm* in DTa1. However, the expression of both *dpp* and *wg* is comparable around Tr1-3 (S1G, H Fig.), arguing against an instructive role of these two factors in *kni* induction in DTa1. Indeed, neither mutants of *tkv*, encoding one of the two *dpp* receptor subunits [68,69] nor *arm* mutants lacking an essential component of *wg* signaling [70–72], showed major defects in *kni* induction and outgrowth of DTa1/CB (S1I, J Fig.).

**Figure 2:**
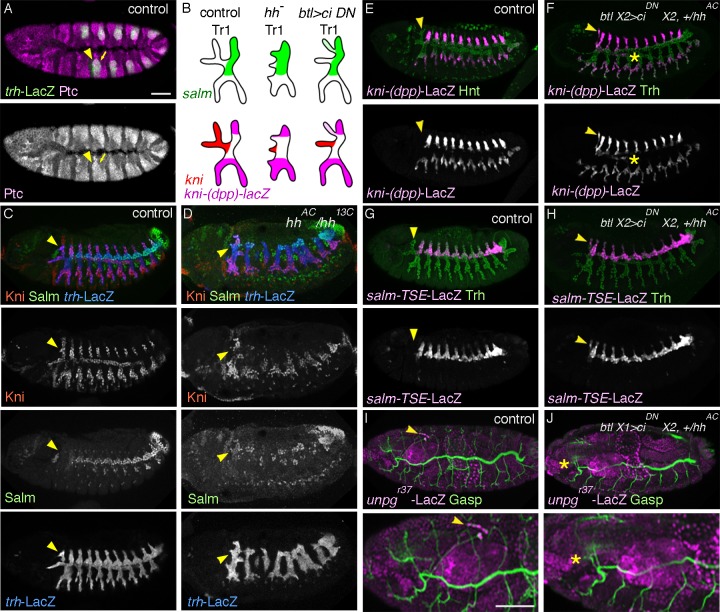
*hh* signaling in the airways is required for DTa1 development. (A) At stage 11, Ptc is detected strongly in all of the invaginated airway primodia (arrow) marked with *trh*-LacZ, with the strongest expression in the anterior part (arrowhead). (B-J) Effects of loss of *hh* signaling on DTa1 development. Arrowheads mark DTa1. A summary of phenotypes is shown in (B). (C, D) Expression of Kni and Salm at stage 13 in relation to the airway development visualized with *trh*-LacZ. In the control (C), DTa1 expresses Kni but not Salm while in *hh* mutants (D) the DTa1 remnant often expresses Salm. Kni is variably detected in these DTa1 remnants. (E-J) Effects of *btl-gal4* mediated overexpression of *ci^DN^* on expression of *kni-(dpp)-lacZ* and *salm-TSE-lacZ*. The airway cells are labeled by Trh or Hindsight (Hnt) [126]. In the control (E,G), *kni-(dpp)*-LacZ (E) is detected in DB, LT and GB while *salm-TSE*-LacZ (G) is detected in DT except for DTa1. Upon overexpression of Ci^DN^ with *btl*-Gal4 (F, H), DTa1/CB additionally becomes positive for respective enhancer reporters. Asterisks mark *kni-(dpp)*-LacZ expression ectopically induced in VB. (I, J) Stage 16 embryos marked with Gasp and *unpg*-LacZ. Parts of each image are enlarged in the bottom. In the control (I), CB forms a slightly thinner branch compared to DB1 and its tips strongly express *unpg*-LacZ. Overexpression of Ci^DN^ variably generates CBs resembling DB1 in branching patterns, tube diameter or lack of *unpg*-LacZ expression (J). Asterisk marks the misrouted CB/DTa1. Scales bars: 50um.

*hh* is a signaling molecule that binds its receptor *patched* (*ptc*), thereby relieving *ptc*-mediated inhibition of the 7 transmembrane domain protein *smoothened* (*smo*) [73,74]. *hh* is expressed in stripes in the ectoderm, abutting the anterior edge of the airway primordia at stage 10 and overlying the anterior part of the invaginated airway cells of each metamere at stage 11 (S1K, L Fig.) [34]. Glazer and Shilo showed that *hh* induces marker gene expression in the anteriorly migrating branches of central metameres [34], arguing that *hh* signaling patterns the anterior primary branch fates of the “typical”, central metameres. We found that just after invagination of the airway primordial cells, expression of *ptc*, a transcriptional target of *hh* [75,76] is upregulated in the DTa1 precursors (Fig. 2A), suggesting that *hh* signaling is active there. In *hh* mutants the dorsalward CB extension is hardly detectable and *salm* expression is expanded in the entire DT1 (Fig. 2B-D). This suggests that *hh* signaling in DTa1 induces *kni* and thereby represses *salm* expression. Among the ectopically *salm* expressing cells in DTa1, some cells also express *kni* while others do not. We suggest that in the absence of *hh* signaling, *hh* responsive *kni* induction is lost while *dpp* signaling may take over to induce *kni* expression in some *salm* positive cells. Such an ectopic activation of *kni* in the absence of *hh* could induce ectopic *kni/salm*-double positive cells resembling DB1 cells. This interpretation is consistent with the de-repression of *kni-(dpp)-lacZ* in *Df(kni/knrl)* mutants (Fig. 1E). Thus, we conclude that *hh* signaling is required to induce *kni* and to repress *salm* in DTa1. The earlier function of *hh* is also required for the maintenance of the striped expression of *wg* [75,77], which induces *salm* expression in DT. Thus, the variability of *salm* expression either in DTa1 or in DTa/DTp of any metamere in *hh* mutants may partly reflect a reduction or loss of epidermal *wg* expression.

In *hh* mutants, *bnl* expression guiding DB migration in central metameres is lost but *bnl* expression in surrounding cells guiding CB is still detectable at stage 12 (S1M Fig.). Nevertheless, the dorsal extension of a CB-like branch is not detected in *hh* mutants at later stages (S1N Fig.). To more directly address the effect of *hh* signaling in DTa1 we attempted to inactivate its components specifically in the airways. *hh* signaling modifies the transcriptional activity of *cubitus interruptus* (*ci*) [74]. In the absence of *hh*, Ci is proteolytically processed and acts as a repressor [78], while upon *hh* pathway activation, *smo* mediated signaling suppresses this proteolysis and turns Ci into an activator [78–80]. The balance of loss of the repressor form and generation of the activator form of Ci determines the *hh* signaling outputs [79–81]. We generated embryos expressing dominant negative forms of *ci, ci^DN^* (*ci^rep^* [82,83] and/or *ci^75^* [78,84]) exclusively in the airways and assessed the expression levels of *kni-(dpp)-lacZ* (a DB and LT/GB marker) [25] and of *salm-TSE-lacZ* (a reporter of *salm* expression) [30] in metamere 1. Both markers are ectopically induced in the DTa1 of these embryos at stage 13 (Fig. 2E-H) although the cell number in this branch did not significantly change (20 cells, standard deviation SD = 0.707 for 5 wild type embryos at stage 13 and 19.2 cells, SD = 0.447 for 5 *btl*X2> *ci^rep^* embryos). We interpret that the incomplete inactivation of *hh* signaling in the airways by *ci^DN^*, partially transformed DTa1 cells to DTp1. These cells are still receiving enough Dpp to express *kni-(dpp)-lacZ*. The weaker effects of *ci^DN^* expressing embryos compared to *hh* mutants may reflect ineffectiveness of Ci^DN^ or the delayed *btl*-Gal4 mediated expression [58] of Ci^DN^, which starts slightly later than the initiation of *hh* action on DTa1. Additionally, the airway-specific overexpression of Ci^DN^ or general *hh* inactivation in *smo* mutants frequently resulted in DTa1/VB1 co-segregation defects and CB misrouting at stage 16 (Fig. 2I, J and S1O Fig.). A similar CB misrouting phenotype has been described in mutants of *unplugged* (*unpg*) encoding a TF expressed in CB [54]. Indeed, the expression of an *unpg* enhancer trap in the CB of wild type embryos is lost upon Ci^DN^ overexpression (Fig. 2I, J).

Collectively, these results identify a selective, direct role of *hh* signaling in inducing the distinct cell identities of DTa1 compared to the cells of the remaining DT branches.

### *hh* pathway overactivation transforms DB1 and DTp1 into DTa1-like branches

**Figure 3:**
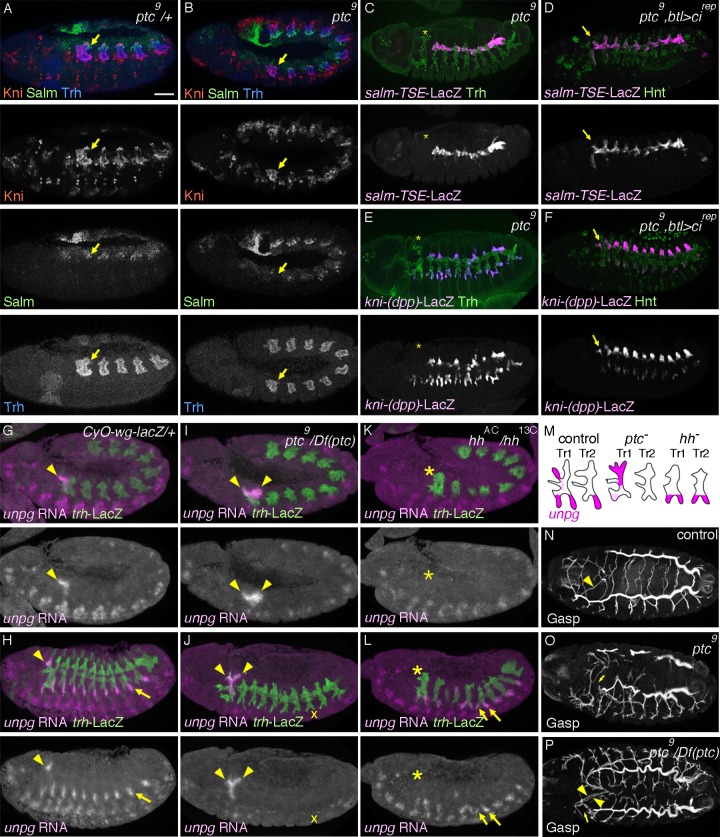
Overactivation of *hh* signaling transforms the primary branch identities in Tr1. (A, B) Expression of Salm, Kni and Trh at late stage 11. In contrast to the control (A), where Salm is upregulated in DTp1 (arrow), in *ptc* mutants (B), Salm upregulation in DTp1 (arrow) is not detected and Kni occupies the whole distal part of Tr1. Note that the airway primordia look smaller in *ptc* mutants [33]. (C-F) Effects of *ptc* mutations on *kni-(dpp)-lacZ* and *salm-TSE-lacZ* expression. The airway cells are labeled by Trh or Hnt. Compared to the control (Fig. 2E,G), in *ptc* mutants (C, E), the expression of *salm-TSE-lacZ* (C) and *kni-(dpp)-lacZ* (E) is lost from the dorsal part of Tr1 (asterisks). Overexpression of Ci^DN^ with *btl*-Gal4 in *ptc* mutants restores their expression in Tr1 (D, F, arrows). (G-M) *unpg* RNA expression at stage 12 (G, I, K) and stage 13 (H, J, L) in relation to the airway development visualized with *trh-lacZ*. A summary of phenotypes is shown in (M). (G, H) In the control, *unpg* RNA is strongly detected in CB (arrowheads) at stages 12 and 13. *unpg* expression is also strong in GB at stage 13 (arrow). (I, J) In *ptc* mutants, *unpg* expression is expanded to DB1, DTp1 and TC1 (arrowheads) while its expression in GB is barely detectable (asterisks). (K, L) In *hh* mutants, *unpg* expression is lost in CB (asterisk) while it is derepressed in LTa in the central metameres (arrows). (N-P) Airway branching pattern visualized by Gasp. Compared to the control (N), *ptc* mutants (O, P) show persistent loss of DT1/2 fusion (asterisks). In addition, duplication of CB like branches occasionally happen (P, arrowheads). Scales bars: 50um.

The transformation of DTa1 to DTp1/DB1 upon inhibition of *hh* signaling suggests that its overactivation may be sufficient to transform DTp1/DB1 to DTa1. To examine this, we analyzed *ptc* mutants, where *hh* signaling is inappropriately activated due to the loss of *ptc*-mediated inhibition of *smo* [73,74]. In *ptc* mutants, the dorsal part of metamere 1 expresses Kni but not Salm already at late stage 11 (Fig. 3A, B). Correspondingly at stage 13, expression of *salm-TSE-lacZ* is specifically lost from metamere 1, suggesting a defect in both DTp1 and DB1 specification (Fig. 3C and S3A Fig., note that in wild type, *salm* is expressed in DB1 and DTp1). Consistent with a loss of the DB1 fate in *ptc* mutants, *kni-(dpp)-lacZ* expression in the dorsal part of metamere 1 is completely lost (Fig. 3E) while Kni protein is expressed in the whole distal part of Tr1 (S2B Fig.). Although *ptc* mutants contain fewer airway cells [33], presumably due to an early upregulation of *wg* [85], a negative regulator of the airway primodia size [86], reduction of cell number in *CycA* mutants [38] does not significantly abrogate DB1/DT1 fates (S2D, H Fig.). This suggests that the effect of *ptc* on DB1/DT1 specification is more direct and not due to a general reduction in the number of airway cells. Because *btl*-Gal4 driven Ci^rep^ (Fig. 3D, F) or Ci^75^ can restore the expression of both *kni-(dpp)-lacZ* and *sal-TSE-lacZ* in the Tr1 cells of *ptc* mutants and because Ptc is expressed in all airway primordia including the entire Tr1 primordium (Fig. 2A), these results suggest that overactivation of *hh* signaling in Tr1 abolishes the DTp1/DB1 fates.

To further test the role of *hh* signaling in determining branch identities in Tr1 we analyzed the RNA expression of *unpg*. In control embryos at early stage 12, *unpg* expression is strongly detected in DTa1 [54] and weakly in the anterior part of TC1. Both of these regions correspond to *hh* signaling activation (Fig. 3G). At stage 13, *unpg* RNA is detected in CB and GB0/GB1 in Tr1 and also in the GBs of the more posterior metameres Tr2-Tr9 (Fig. 3H) [54]. Consistent with the loss of *unpg-lacZ* expression in CB upon Ci^DN^ overexpression, *unpg* RNA expression is lost in DTa1/CB of *hh* mutants (Fig. 3K, L). Conversely in *ptc* mutants, it is expanded posteriorly to cover the positions of DB1/DTp1/TC1 (Fig. 3I, J), indicating their transformation to CB-like fates. Consistent with the expanded *unpg* expression, we often detected a duplication of CB-like branches in *ptc* mutants (Fig. 3P). We additionally noted that *unpg* expression in GB is lost in *ptc* mutants (Fig. 3J) while *unpg* is derepressed in LTa in *hh* mutants (Fig. 3L), in accord with the notion that *hh* confers the anterior branch identity in the central metameres [34].

At stage 16, *ptc* mutants show variable branching defects including stalled GBs, DBs and DT breaks [33]. Concomitantly with the loss of DTp1 fate marker (*salm-TSE-lacZ*), DT1 and DT2 never fuse in *ptc* mutants (Fig. 3N-P). In wild type, one of DTp1 cells takes the fusion cell fate, activates *dys* expression and attaches to a fusion cell in DTa2 (S2E Fig.), [18]. In *ptc* mutants, *dys* is not activated in DTp1 while *dys* expression is variably expanded in more cells of the DT branches in posterior metameres (S2E-G Fig.). This may reflect an increase of epidermal expression of *wg* [85], an inducer of the fusion cell fates [27,28]. The failure of *dys* activation and DT1 fusion in *ptc* mutants is significantly rescued by *btl*-Gal4 mediated overexpression of Ci^rep^ (S2I Fig.). Moreover, both loss of *dys* expression in DT1 as well as DT1/2 fusion defects are variably observed when dominant active Ci, Ci^act^ [84,87] is overexpressed in the airway cells (S2J Fig.). Notably however, the Ci^act^ overexpression by *btl*-Gal4 did not diminish *salm-TSE-lacZ* or *kni-(dpp)-lacZ* expression.

In summary, we suggest that *hh* signaling instructs the DTa1 fate at the expense of DB1/DTp1 fates. In DTp1, *hh* signaling must be kept low to allow the proper selection of the fusion cell fate and subsequent DT1/DT2 branch fusion.

### Abdominal Hox genes shunt *hh* signaling from induction of *kni* in DTa

The *hh* induction of *kni* expression in the DTa of wild type embryos as well as the loss of DT/DB fates in *ptc* mutants are confined to Tr1. However, *hh* signaling outcomes are expected to be equally profound in the more posterior metameres of both wild type [34] and *ptc* mutant embryos [33]. The exclusive restriction of Hh responses within Tr1 implies the presence of an inhibitory mechanism preventing *kni* activation in the DTa branches of posterior metameres. The BX-C genes represent obvious candidate regulators of posterior metamere identity and modulators of *hh* signaling outcomes along the A-P axis of the airways. Indeed, *unpg* expression is de-repressed in progressively more posterior metameres in *Ubx* mutants and *Ubx abdA AbdB* triple mutants [54].

BX-C gene expression is graded along the A-P axis of the airway metameres (S3A-D Fig.). *Ubx* expression starts in Tr2 (PS5) and peaks at Tr3 (PS6) (S3A Fig.). *abdA* expression starts in Tr4 (PS7) and peaks in Tr6 (PS9) (S3B Fig.) while *AbdB* expression starts in Tr7 (PS10) and peaks in Tr10 (PS13) (S3C Fig.). These expression patterns are in register with the expression of BX-C genes in the ectoderm [53], which is the origin of the airway primordia.

**Figure 4:**
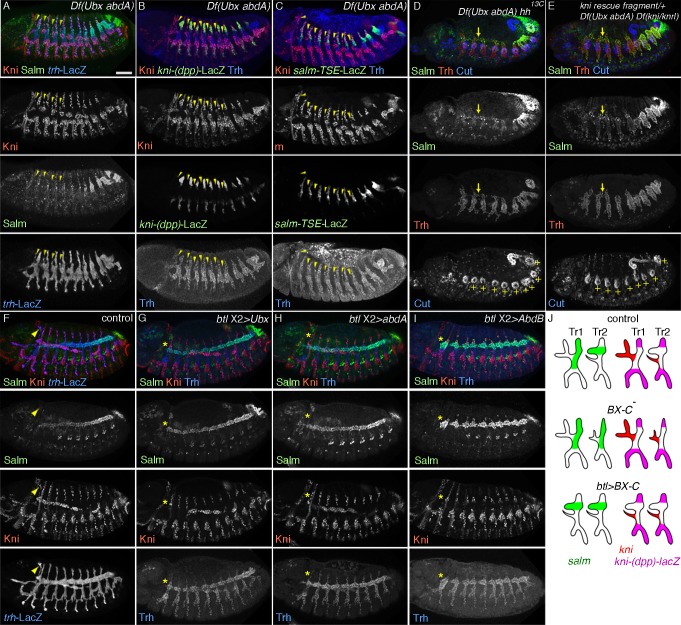
The airway intrinsic BX-C code determines the outcome of *hh* signaling. (A-C) Effects of loss of *Ubx* and *abdA* on the DTa fate in the central metameres. In *Df(Ubx abdA)* mutants at stage 13, ectopic dorsalword migration of cells that are positive for Kni and negative for Salm is variably detected in metameres 1–7 (A). These transformed DTa/CB-like cells (arrowheads) are negative for both *kni-(dpp)-lacZ* (B) and *salm-TSE-lacZ* (C). Note that expression of Salm/*salm-TSE*-LacZ is weakly detected in DB, DTp and TC in the transformed metameres as well as in Tr1. (D, E) Effects of *hh* or *kni/knrl* mutations on Salm expression in DTa of *Df(Ubx abdA)* mutants. Compared to *Df(Ubx abdA)* mutants (A-C), in *hh Df(Ubx abdA)* (D), or in *Df(kni/knrl) Df(Ubx abdA)* mutants, where early gap gene function of *kni* is rescued (E), Salm expression is equally detected in DTa and DTp. Note that the fusion of DT branches in the central metameres is weakly restored (arrows). This rescue is not due to an artificial collapse of the airway primordia because many of the ectopic Cut positive anterior spiracle (ASP) [125] (+, bottom panels) at the proximal part of the airway remain separate. (F-I) Effects of overexpression of BX-C genes on expression of Salm and Kni. In the control at stage 13 (F), CB (arrowhead) is positive for Kni and negative for Salm. Note that Salm expression in DT shows a posterior-to-anterior decrease. Upon overexpression of *Ubx* (G), *abdA* (H) or *AbdB* (I), DTa1 becomes negative for Kni and positive for Salm (asterisks). Note that the posterior-to-anterior decrease of Salm expression is variably disrupted, most prominently upon *AbdB* overexpression. Note that DB in all metameres strongly expresses Salm upon *AbdB* overexpression. (J) A summary of BX-C mutant conditions regarding primary branch identities. Scales bars: 50um.

To explore the function of BX-C genes in DTa fates, we first monitored *dys* expression in various BX-C mutants. In *Ubx* mutants, single fusion cells are detected in Tr1, Tr2 and Tr3 (S3E, F Fig.) suggesting that DTa2 and 3 are transformed to become DTa1/CB. *abdA* single mutants do not show *dys* expression defects in the DT (S3G Fig.) while a superfluous fusion cell in DTp10 is detected in both *AbdB* single and *abdA AbdB* double mutants (S3H, I Fig.). This suggests that DT10, which normally contains only a single fusion cell in its DTa branch, is transformed into a more anterior identity. Compared to *Ubx* single mutants, *Ubx abdA* double mutants have single fusion cells in Tr1–8 and often in Tr9 (S3J Fig.). In *Ubx abdA AbdB* triple mutants, the DT stumps of all metameres contain single fusion cells (S3K Fig.). This implies that DTa branches in progressively more posterior metameres are transformed to become DTa1 upon progressive loss of BX-C genes [39]. Any single gene of the BX-C is sufficient to suppress the DTa1 fate. Consistently, we detected expansion of *kni* expression and a loss of *salm* in the transformed DTa in *Ubx* single, *Ubx abdA* double and *Ubx abdA AbdB* triple mutants (Fig. 4A-C). These phenotypes are often accompanied with the appearance of dorsally extending branches that are positive for Kni but negative for *kni-(dpp)*-LacZ, Salm and *salm-TSE*-LacZ, resembling the CBs of wild type embryos (S3K Fig.) [54]. The marker expression analysis in BX-C mutants suggests that in the posterior metameres, *Ubx, abdA* and *AbdB* interfere with the outcomes of *hh* signaling.

If the antagonistic role of BX-C on *hh*-mediated *kni* induction reflects an essential function of the BX-C in posterior metameres, one might expect some rescue of the branching defects of BX-C mutants upon simultaneous loss of *hh* or *kni/knrl*. Indeed, *salm* expression is de-repressed in DTa1-9 of *Ubx abdA hh* and of *Ubx abdA kni/knrl* mutants (Fig. 4D, E). Additionally, DT fusion is weakly restored in both the triple and quadruple mutants (S3L, M Fig.).

Taken together, we suggest that BX-C genes antagonize *hh*-mediated induction of *kni* in DTa branches.

### Airway-specific expression of abdominal Hox genes diverts *hh* signaling from *kni* activation

In addition to the airways, BX-C genes are expressed in many embryonic tissues. Where do they act to divert *hh* signaling from *kni* induction in the DT branches? In lack of reagents for the reliable conditional inactivation of the BX-C genes in the airways, we monitored the effects of airway-specific ectopic expression of BX-C genes on DT1 cell specification. The first metamere does not express the BX-C genes (S3A-D Fig.) and thereby may provide a naïve environment for assessing the effects of their overexpression on marker gene activation [41]. *btl*-Gal4 mediated overexpression of any of the BX-C genes in wild type background, variably decreases *kni* expression in the DTa1 and concomitantly leads to increased *salm* levels at stage 13/14 (Fig. 4F-J). At later stages, DTa1 branches are thick, resembling typical DTa branches of posterior metameres (see below) in agreement with the previously reported loss of CBs upon *abdA* overexpression [88].

Similarly, *btl*-Gal4 mediated overexpression of either *Ubx* or *abdA* restores the fusion defects of DT1 and DT2 in *ptc* mutants (S3N, O Fig.). We detected that expression of both *salm-TSE-lacZ* and *kni-(dpp)-lacZ* is restored in the dorsal part of Tr1 of *ptc* mutants upon *abdA* overexpression (S3P, Q Fig.). These results argue that the Hox code in the airway cells autonomously changes *hh*-signaling outputs in Tr1 both in wild type and in *ptc* mutants, where the *hh* pathway is hyper activated. Finally, we asked if transgenic expression of *abdA* or *Ubx* in the airways could restore the branch fusion defects along the entire DT of *Ubx abdA* double mutants. Again, this manipulation rescues the branch fusion phenotypes (S3R-T Fig.) arguing that BX-C genes autonomously shunt *hh* signaling from inducing *kni* in the DTa branches of all metameres to promote continuous DT formation.

### BX-C genes control tube tapering

A common characteristic of biological tubes is the tapering of tube diameter along their length. The *Drosophila* larval airways receive air only from the PSP and distribute it anteriorly. Correspondingly, the tubes show a posterior to anterior tapering [38], which presumably gradually increases the flow rates to the anterior and facilitates air diffusion from the PSP to the most distant anterior organs (http://hyperphysics.phy-astr.gsu.edu/hbase/pfric.html).

*salm* is a master selector gene for DT identity. Intriguingly, its expression levels in the DT tubes at stage 13/14 show a largely proportional decrease from posterior to anterior metameres matching the tapering of the airways (Fig. 4F)[30]. The graded diameter along the airway length also coincides with the graded expression of BX-C proteins along the A-P axis. To explore the potential regulatory roles of BX-C factors and *salm* in tube shaping, we first analyzed airway shapes in BX-C mutants and detected 2 kinds of effects of BX-C genes on tube diameter, metamere-autonomous and systemic (see below).

**Figure 5:**
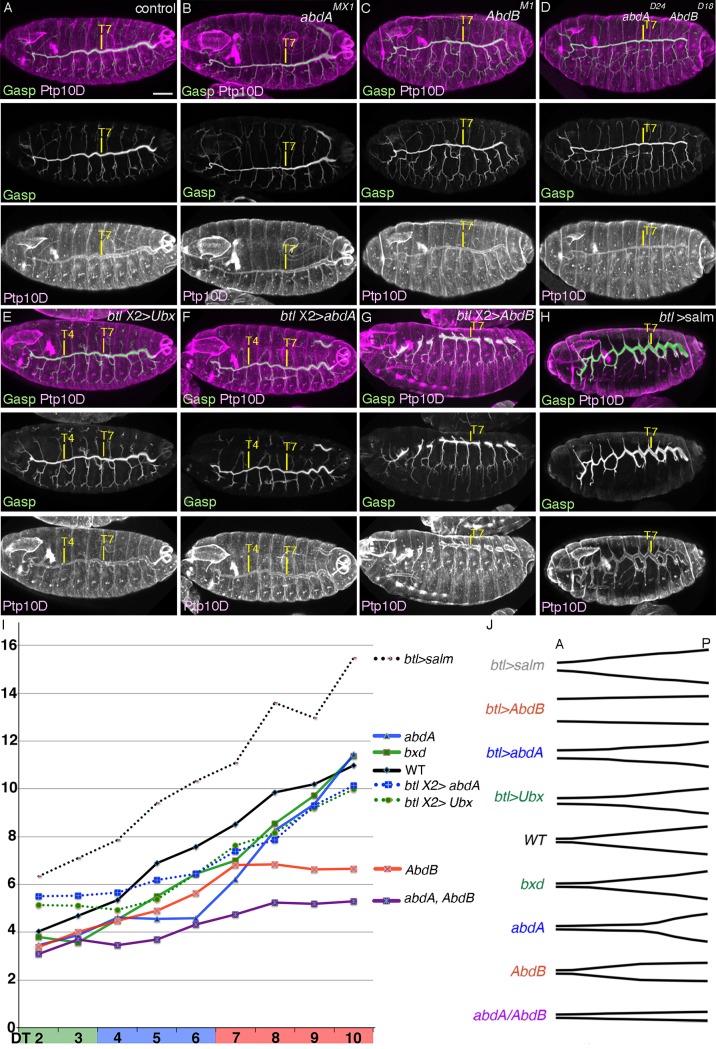
BX-C genes control DT tube tapering. (A-H) BX-C control of DT tube tapering visualized by luminal Gasp and the apical protein PTP10D. In the control (A), DT tube diameter shows posterior-to-anterior tapering. In *abdA* mutants, tube diameter of DT4-6 approaches that of DT3 while in *AbdB* mutants, tube diameter of DT7-10 resembles that of DT6. In *abdA AbdB* double mutants, tube diameter of DT4-10 resembles that of DT3. Note that there is still a weak tapering of the tube. Upon overexpression of *Ubx* (E) or *abdA* (F), tube diameter of DT2/3 becomes thicker than the control. We noticed that DT diameter in the posterior metameres becomes adversely reduced. This could be attributed to competition between the overexpressed proteins and endogenous *abdA* or *AbdB* [46], leading to a systemic decrease of tube diameter. Upon overexpression of *AbdB* (G), DT fusion becomes frequently defective. However, it is apparent that DT tube diameter of the anterior metameres resembles that of DT10. Upon overexpression of *salm* (H), DT tube diameter becomes thicker than the control. In addition, tube diameter of other branches also becomes thicker [28]. (I,J) Quantification (I) and cartoons (J) of diameter phenotypes of this figure and other figures are shown. Raw data used for quantification and some statistics are shown in S1 Table. See text for details. Scales bars: 50um.

Consistent with graded *AbdB* expression in PS 10–13, in *AbdB* mutants, tube diameter in DT7-10 lost its tapering and became narrower suggesting that the amount of *AbdB* proportionally controls the tube diameter. (Fig. 5A, C, I, J and S1 Table). Similarly, in *abdA* mutants, the gradient of tube diameter in DT4-6 was lost and DT4-9 became narrower (Fig. 5B, I, J and S1 Table) suggesting again that AbdA levels proportionally control DT tube diameter. In *abdA AbdB* double mutants the shape of the airways is changed further (Fig. 5D, I, J and S1 Table). The airways of Tr4-10 acquire a more cylindrical shape compared to the conically shaped tubes of wild type embryos. We suggest that in wild type embryos the gradient of *abdA* and *AbdB* activities would superimpose on a weak but clear, *abdA* and *AbdB*-independent gradient of DT tube thickness (Fig. 5I and S1 Table). This may explain why *abdA* mutants, where PS7-9 (DT4-6) are expected to transform to PS6 (DT3) still show a clear difference in tube diameter between DT3 and DT4 and why *AbdB* mutants, where PS10-13 (DT7-10) are expected to transform to PS9 (DT6) show a distinct tube caliber in DT6 and DT7 (Fig. 5I and S1 Table). Thus, in accord with their known functions in determining cell fates and morphogenesis in the embryonic ectoderm [39,44,53], the BX-C genes control the tapering of airways along the A-P axis autonomously. We note however that there is also a systemic effect of BX-C mutations along the entire DT. In either *abdA, AbdB* single or in *abdA AbdB* double mutants, the diameter of the more anterior metameres, where corresponding BX-C genes are not expressed also show a slight reduction of tube diameter (Fig. 5I and S1 Table). Among different possibilities, these results may suggest that the activities of *abdA* or *AbdB* control the hydrostatic pressure in the lumen to non-autonomously assure proportional growth of all the DT tubes [89] (see below). The residual tapering of *abdA AbdB* double mutants implies a mechanism of A-to-P gradient formation independent of *abdA* and *AbdB. Ubx* could exert such a function in the absence of *abdA* and *AbdB*. We analyzed *bxd^113^* or *bxd^100^* mutants, where Ubx expression levels in PS5 become similar to that of PS4 but its ectodermal expression is lost from PS7 onwards [90] (S4A Fig.). In these embryos, DT3 tube diameter approaches that of DT2 and there is also an overall reduction of tube diameter in more posterior metameres (Fig. 5I, S4C-E Fig. and S1 Table), suggesting that the endogenous Ubx level controls DT diametric tube expansion. However, in *abdA AbdB* double mutants, Ubx levels are largely uniform in DT4-10, which correspond to PS7-13 [91] (S5B Fig.). This suggests the presence of an additional, BX-C-independent cue in DT tube shaping. In *Ubx abdA AbdB* triple mutants, the transformed Salm positive residual DT/TC branches in posterior metameres are slightly thicker than those in anterior metameres, further arguing for the existence of the postulated BX-C-independent mechanism in tube shaping (S3K, S4F Figs.).

Is there any causative link between the BX-C mediated DT expansion control and the A-to-P gradual increase of *salm* expression levels in the DT (Fig. 4F) [30]? We noticed that Salm levels are reduced in central and posterior metameres of *abdA, AbdB* or *abdA AbdB* double mutants (S4G-J Fig.). This reduction is largely consistent with the changes in shape and DT tube diameters in the corresponding mutants. Conversely, upon overexpression of *AbdB*, higher Salm amounts are detected in the DT of all metameres at stage 13 (Fig. 4I). The DT branches of these embryos often stall and fail to fuse making evaluation of tube diameter difficult. Nevertheless, the DT diameter in all metameres appears comparable to the diameter of the most posterior DT branches (Fig. 5G). Overexpression of *Ubx* or *abdA* renders Salm expression levels uniform in the anterior metameres (Fig. 4G, H). Correspondingly, the DT diameter in the anterior metameres becomes thicker (Fig. 5E, F, I, J and S1 Table).

How does the Salm gradient along the DT A-P axis correlate to the graded DT tube expansion? *salm* overexpression confers DT identity to other primary branches [28,62]. We noted that *salm* overexpression also generally expands tube diameter not only in the transformed branches [28] but also in the DT, which endogenously expresses *salm (* Fig. 5H, I, J and S1 Table). The programmed secretion of luminal and apical proteins has been proposed to drive tube dilation of the *Drosophila* airways [92–95]. Tenectin (Tnc) is a luminal glycoprotein accumulating in the DT and hindgut tubes during diametric expansion [96]. Tnc overexpression in the airways drives DT tube dilation in a dose-dependent manner potentially through increasing hydrostatic pressure [89] and the *tnc* mRNA levels increase in a characteristic graded fashion along the A-P axis of the DT tubes in wild type embryos [89]. We found that the diametric increase caused by *salm* overexpression in the airways is accompanied by an increase in the luminal levels of Tnc and conversely, Tnc becomes undetectable in the tracheal tubes of *salm* mutants (S4K-M Fig.). This suggests that Salm adjusts the graded expression levels of Tnc and presumably other proteins during tube dilation.

Additionally, the diameter increase in the branches of *salm* over expressing embryos is still most pronounced in the posterior metameres. (Fig. 5H, I, J and S1 Table). The accentuated tube enlargement in posterior metameres upon *salm* overexpression suggests a *salm*-independent control mode of A-to-P gradient of DT tube diameter. This is consistent with the observation that tube diameter of the remaining branches in *salm* mutants are still thickest in Tr10 (S4N Fig.).

**Figure 6:**
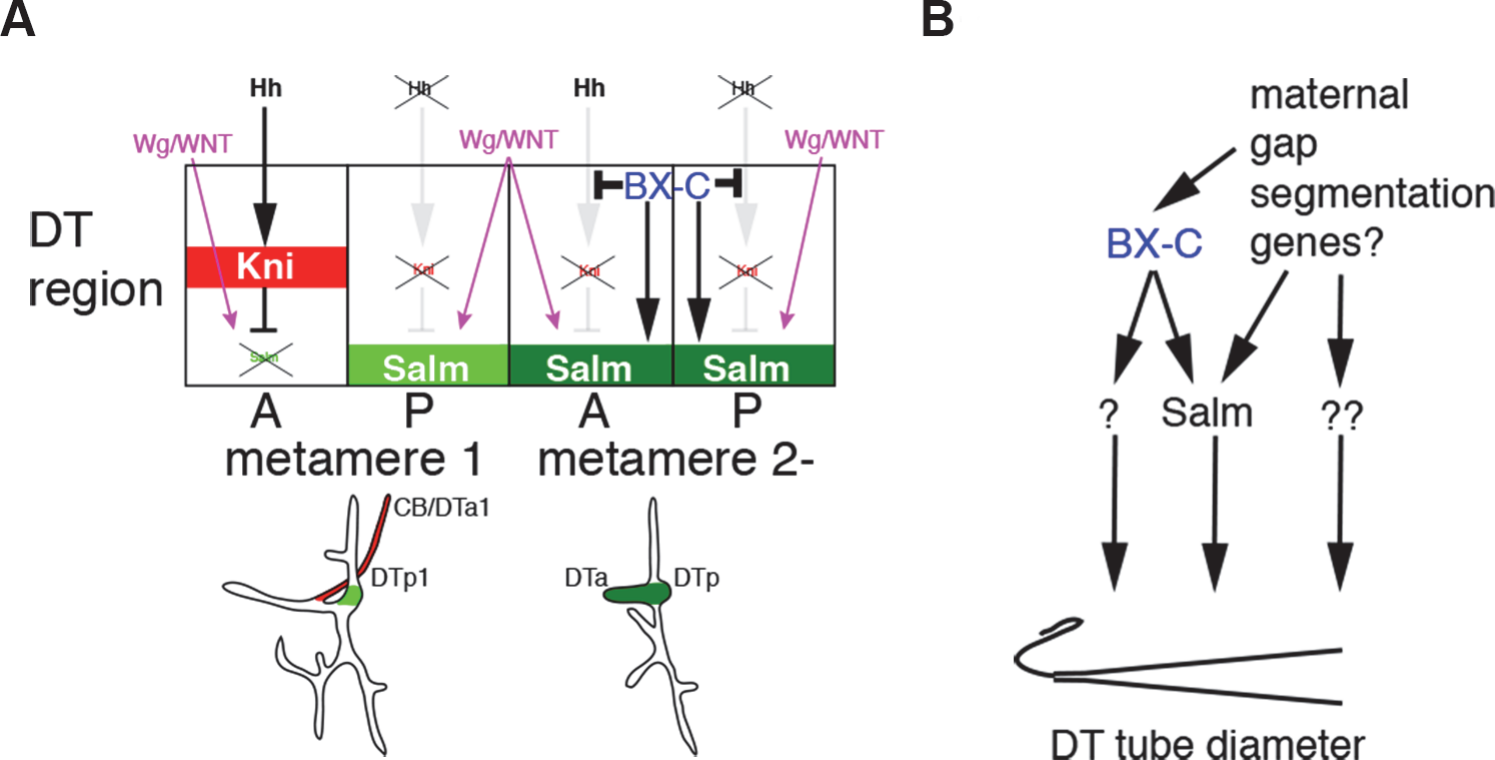
Models for the BX-C dependent control of branching patterns and of DT tube diameter. (A) In all metameres, expression of *hh* and *wg* in cells surrounding the tracheal primordia are comparable. In metamere 1, where there is no input from BX-C, *hh* signaling induces *kni* in DTa1 in wild type, and additionally in DTp1 in *ptc* mutants. *kni* represses *wg/WNT* mediated upregulation of *salm*, making DTa1 branches thinner. In the posterior metameres, BX-C shunts *hh* signaling from induction of *kni*, allowing *salm* upregulation by *wg/WNT*. BX-C additionally enhances *salm* expression in the DT. (B) BX-C genes coordinate the posterior-to-anterior tube tapering through *salm* dependent and independent modes. DT tube tapering may also be regulated independently of BX-C genes through inputs from the maternal, gap or segmentation gene cascades.

In conclusion, our work suggests two interdependent mechanisms by which the tube intrinsic Hox code controls branch identity and tube shape in the *Drosophila* respiratory network (Fig. 6A, B). Firstly, Hox genes autonomously divert extrinsic *hh* signaling from *kni* induction in the DT thereby generating continuous, *salm* positive DT airways. We suggest that this allows a second tier of DT tube shape regulation, where the Hox activity gradient both locally and systemically organizes graded tube dilation via *salm* dependent and independent modes. The Ci/BX-C circuit may control tube morphology directly by binding to the regulatory regions of *kni* and *salm*, as recent genome-wide TF binding studies suggest that *kni* is a direct target of *ci* [37] and that *salm* is a direct target of *Ubx* [97]. In our model, tube tapering and thereby luminal fluid flow are calibrated by the balance between extrinsic signals and the intrinsic Hox code. Since Hox selector genes are regionally expressed in other developing tubular organs like the mammalian lung [98] and vasculature [9,10], a similar regulatory logic of tube branching and shaping may apply to other systems.

## Materials and Methods

### Fly genetics

Flies kept over balancer chromosomes [99] were grown in standard medium. We obtained the appropriate genotypes by standard genetic crosses. For overexpression of genes, we used the Gal4/UAS system [100]. Mutant embryos were identified by the expression of *twi-lacZ, ftz-lacZ, Ubx-lacZ or dfd-GFP* [101] constructs inserted on balancer chromosomes. We identified mutants harboring *Ubx* or *AbdB* mutations by selecting embryos with previously reported phenotypes in the anterior spiracle or PSP. For collection of large amount of virgins, we used a Y chromosome harboring *hs-hid* construct developed by R. Lehmann and M. Van Doren [102]. See Flybase [103] for details of strains described below.

**Mutant strains.** *abdA^M1^* (a gift from B. Gebelein) [104], *AbdB^M1^, AbdB^M5^* and *Df(3R)P115* as *Df(Ubx abdA AbdB)* (gifts from I. Lohmann) [105], *btl^∆oh10^* and *btl^∆oh24-1^*[106], *Df(5)* as *Df(salm/salr)* (a gift from M. Llimargas) [28,107], *hh^13C^* [108], *kni early rescue fragment; Df(3L)riXT* (a gift from R. Schuh) [25], *rho^d38^* (a gift from D. Andrew and A. Salzberg) [109,110], *rho^7M^* (a gift from J. Skeath) [111], *top^f24^* (a gift from K. Moses) [112]. *arm^4^* and *CycA^3^* were obtained from Natinal Institute of Genetics (NIG), Mishima, Japan. *Ubx^1^, abdA^MX1^, abdA^D24^ AbdB^D18^, Ubx^1^ abdA^D24^ AbdB^D18^[113], Df(109)* as *Df(Ubx,abdA), CycA^C8LR1,^ Df(3L)Exel6115* as *Df(CycA), hh^AC^, Df(3L)BSC448* homozygous or its transheterozygote over *Df(3L)riXT* as *Df(kni/knrl), ptc^9^*, *Df(2R)Exel7098* as *Df(ptc), smo^3^, tkv^7^* and *Df(2R)Exel6076* as *Df(top)* were obtained from Bloomington stock center (BDSC), Indiana, USA.

**Enhancer trap strains.** *1-eve-1* as *trh-lacZ* (a gift from N. Perrimon) [114] and *unpg^r37^* (a gift from F. J. Diaz-Benjumea, R. Urbach and G. M. Technau) [115,116]. *hh^P30^* [117] was obtained from BDSC.

**Enhancer reporter strains.** *kni-(dpp)-lacZ* [25] and *salm-TSE-lacZ* [30] (gifts from R. Schuh).

**Gal4 and UAS strains.** *btl-gal4* on 2^nd^ and 3^rd^ chromosomes (gifts from S. Hayashi) [58], *UAS-abdA* (a gift from F. J. Diaz-Benjumea) [115], *UAS-ci^75^* and *UAS-ci^H4P^* (gifts from S. Ishii) [84], UAS-ci^rep^ (a gift from A. Moore) [83], *UAS-kni* and *UAS-knrl* (gifts from R. Schuh) [25]. *UAS-AbdB, UAS-Axn-GFP, UAS-salm* and *UAS-Ubx* were obtained from BDSC.

### In situ hybridization and immunostaining

Egg collection was done with apple/grape juice plate at 25°C. Embryos were bleached and fixed as previously described [118] for 15–30 minutes with a 1:1 mixture of heptane and a fix solution (3.7% formaldehyde, 0.1M Hepes pH6.9, 2mM MgSO4). Embryos were dechorionated with methanol and incubated in 0.1% PBT supplemented with 0.5% BSA. Staging of embryos was done as previously described [119].

For immunostaining the following primary antibodies were used:

Guinea-pig anti-AbdA (1:500, a gift from B. Gebelein) [104], rabbit anti-Dys (1:500, a gift from L. Jiang) [18], Guinea-pig anti-Gasp (1:1000) [95], mouse anti-Ubx (1:10, a gift from R. White) [120], Guinea-pig anti-Kni (1:300), (developed by J. Reinitz and distributed by Y. Hiromi, East Asian Segmentation Antibody Center, Mishima, Japan) [121], rabbit anti-Salm (1:200, gifts from R. Barrio and T. Cook) [107,122], rabbit anti-Tnc C-terminal (1:1000, a gift from Z. A. Syed and A. Uv) [89], rat anti-Trh (1:200, a gift from D. Andrew) [123] and rabbit anti-Trh (1:50). Mouse anti-Abd-B (1:10, donated by S. Celniker) [124], mouse anti-Cut (1:10, donated by G. M. Rubin) [125], mouse anti-Hnt (1:10, donated by H. D. Lipshitz) [126], mouse mab2A12 (anti-Gasp) (1:5, donated by M. Krasnow, N. Patel and C. Goodman) [17,95], mouse anti-Ptc (1:10, donated by I. Guerrero) [127] and mouse anti-PTP10D (1:10, donated by K. Zinn) [128] were obtained from Developmental Studies Hybridoma Bank (DSHB), Iowa, USA. Commercially available antibodies were anti-LacZ (*E.coli*. β-Galactosidase) antibodies made in goat (1:500, Biogenesis) or rabbit (1:1000, Capel) and anti-GFP antibodies made in rabbit (1:500, JL-8 Clontech), mouse (1:1000, GFP20 Sigma) or goat (1:500, ab6673 Abcam).

Donkey or goat biotin- or fluorescently labeled secondary antibodies made against the host species of primary antibodies were purchased from Jackson Laboratories. Streptavidin coupled with AMCA, FITC or Cy5 were used when necessary. For mab2A12 detection TSA amplification (PerkinElmer) was used.

Double fluorescent labeling with RNA probe and antibody was carried out as described [129]. The following cDNA clones were used to make hybridization probes; *bnl* (a gift from M. Krasnow) [20], *dpp* [108] and *unpg* (a gift from P. A. Beachy) [54]. *wg, salm* and *kni* clones were obtained from Drosophila Genomics Resource Center (DGRC), Indiana, USA.

Confocal images were taken by Biorad MRC1024, Oympus Fluoview 1000 or Zeiss LSM780. Images of controls and mutants taken by the same confocal microscopes were used for comparison. Images were processed by ImageJ and figures were prepared with Photoshop and Illustrator.

Quantification of Trh positive cell number of CB was done for stage 13 embryos stained with antibodies against Trh and DE-cad, scanned at 40× magnification with 0.6 um intervals.

For quantification of DT tube thickness, mid-late stage 16 embryos stained with antibodies against PTP10D, Gasp and DE-cad were scanned at 40× magnification with 0.6 um intervals. Using ImageJ, 3 points next to the bases of DB were selected from each Z-stacked image for each metamere to measure the maximal distance of PTP10D positive apical membranes perpendicular to the longitudinal tube axis. 3 embryos for each genotype were measured. Average, SD and p-value of student t-test were calculated by Excel.

## Supporting Information

S1 Fig Expression or potential functions of *dFGF/bnl, dEGFR, wg, dpp* and *hh* for DTa1/CB formation. DTa1 is marked with arrowhead or asterisks in the different panels.
(A-D) *bnl* signaling.
(A, B) Expression of *bnl*.
(A) At late stage 11, corresponding to the 6 primary branches of the airway (marked with *trh-lacZ*), 6 patches of surrounding cells express *bnl* for Tr1.
(B) At stage 12, DTa1 appears to extend toward *bnl* expression corresponding to the DB0 position.
(C, D) Expression of *kni* RNA.
Compared to the control (C), in *btl* mutants (D) at early stage 12, *kni* induction in DB is variably abolished while its DTa1 expression is comparable. Different magnification was used to image the same embryo in Fig. 1B and S1C Fig.
(E-F) *dEGFR* signaling.
In either *rho* mutant (E) or *dEGFR* mutant (F), Kni expression in DTa1/CB at stages 13–14 is comparable to the control (Fig. 1D).
(G-J) *wg* and *dpp* signaling.
*wg* RNA distribution (G) relative to DT or *dpp* RNA expression (H) in the dorsal and the lateral ectodermal stripes at stages11/12 are comparable in all metameres.
(I) In *arm* mutants at stage 13, expression of Kni as well *as kni-(dpp)*-LacZ in DB/LT/GB are comparable to the control (Fig. 1D).
(J) In *tkv* mutants at stage 13, *kni-(dpp)*-LacZ expression in DB/LT/GB is variably abolished while Kni expression in DTa1/CB is comparable to the control (Fig. 1D).
(K-O) *hh* signaling.
(K, L) Exterior (K) or interior (L) sections of Hh expression monitored with a *hh* enhancer trap line. *hh* is detected just above the anterior part of the invaginated airway primodia for all metameres (marked with Trh).
(M) In *hh* mutants at stage 12, *bnl* expression corresponding to DB extension is significantly decreased (cross). The ectodermal patches of *bnl* expression corresponding to DT extension become continuous.
(N, O) Gasp positive airway branching in *hh* or *smo* mutants.
(N) *hh* mutants have variable defects including loss or stalling of branches (DB, DT, VB or CB) or loss of metameres. CB does not form dorsal extension. (O) *smo* mutants often show CB misrouting phenotypes.
Scales bars: 50um.
(TIF)

S2 Fig Effects of *hh* signaling overactivation on airway branching. (A-D) Effects of *ptc* or *CycA* mutations on expression of *kni* or *salm*. A summary of *ptc* mutant phenotypes is shown in (C).
(A, B) In *ptc/Df(ptc)* mutants, expression of *salm-TSE-lacZ* (A) and *kni-(dpp)-lacZ* (B) is lost in the dorsal part of the metamere 1 (asterisks) while Kni protein is expressed in the whole distal trachea in Tr1 (B, bottom panel).
(D) In *CycA* mutants, where the cell number in the airways is reduced nearly to half [38], DBs in all metameres express *kni-(dpp)-lacZ* comparably (arrows) and CB formation can occur (arrowheads).
(E-J) Stage 14–15 embryos stained with Gasp and Dys.
In the control (E), the fusion point of DT1/2 is occupied by two fusion cells originating from DTp1 and DTa2 (arrow).
In *ptc* mutants (F, G), *dys* expression is not detected in the positions of DT1p (and LT1p) (asterisks) while *CycA* mutant embryos (H) are comparable to the control (arrow).
*btl*-Gal4 driven Ci^rep^ (I) restores Dys positive fusion cells in DTp1 as well as fusion of DT1/2 (arrow).
Overexpression of Ci^act^ with *btl*-Gal4 (J) can mimic the phenotypes of *ptc* mutants. Dys expression in DTp1 position is lost and is accompanied by fusion defects of DT1/2 (asterisk).
Scales bars: 50um.
(TIF)

S3 Fig Expression and functions of BX-C Hox genes in relation to airway development. (A-D) Expression of BX-C genes at stage 13 visualized by the respective antibodies. A summary of the expression patterns is shown in (D).
(A) *Ubx* expression starts from metamere 2 and peaks at metamere 3. From metamere 3 to 10, *Ubx* expression forms a gradient.
(B) *abdA* expression starts from metamere 4 and peaks at metamere 6. From metamere 7 to 10, *abdA* expression forms a gradient.
Only the anterior half of metamere 10 expresses *Ubx* and *abdA*.
(C) *AbdB* expression starts in metamere 7 and peaks at metamere 10, forming a single gradient.
(E-K) Airway branching phenotypes of BX-C mutants assessed at stage 15 with expression of Gasp, Dys and Cut.
In the control (E), DT is continuous with a pair of Dys positive fusion cells at each fusion point (arrowhead for DT1/2). The terminal parts of the airway are plugged with Cut positive ASP and PSP (arrows) [125].
In *Ubx* mutants (E), metameres 2/3 are transformed to metamere 1 as judged by the loss of fusion cells at the position of DTa (asterisks) and gain of Cut positive ASP (marked with +).
While *abdA* mutants (F) are largely normal, both *AbdB* single mutants (H) and *abdA AbdB* double mutants (I) have an ectopic fusion cell in DTp10 (asterisks), in addition to loss of Cut positive PSP (crosses).
In *Df(Ubx abdA)* mutants (J), in addition to metameres 2/3, metameres 4–6 are transformed to Tr1 while transformation is variable in Tr7-9. Namely, Dys-positive fusion cells at the position of DTa are lost in Tr2-8 while Tr 9 has 1–2 fusion cell. Fusion of DT9/10 is frequently observed. Cut positive ectopic ASPs are detected in Tr2-9 (marked by +). DTp diameter in Tr7-10 looks thicker compared to the more anterior metameres.
In *Ubx abdA AbdB* triple mutants (K), all metameres are transformed to Tr1. Cut-positive PSP is lost (cross). Instead, Cut positive ASP (+) as well as loss of fusion cell fates at the position of DTa (asterisks) is observed in all metameres. Note that in *Ubx, Df(Ubx abdA)* and *Ubx abdA AbdB* mutants, CB-like branches are variably detected in the transformed metameres [54].
(L, M) Effects of *hh* or *kni/knrl* mutations on DT of *Df(Ubx abdA)* mutants.
In *hh Df(Ubx abd)* mutants (L) or in *Df(kni/knrl) Df(Ubx abdA)* mutants, DT fusion is partially restored (arrows). Note that this rescue is not due to a fusion of the airway primordia because many of the ectopic Cut positive ASPs at the proximal part of the airway are separate.
(N-Q) Effects of overexpression of *Ubx* or *abdA* on *ptc* mutant phenolypes in Tr1.
Compared to *ptc* mutants (S2 Fig.), ectopically supplied *Ubx* (N) or *abdA* (O) in the airway cells significantly restores both fusion cell fates in DTa1 and subsequent fusion of DT1/2 (arrowheads). Similarly, *abdA* overexpression in *ptc* mutants restores expression of *kni-(dpp)-lacZ* (P) and *salm-TSE-lacZ* (Q) to metamere 1.
(R-T) Effects of overexpression of *Ubx* or *abdA* to the airway branching of *Ubx abdA* double mutants.
In *Df(Ubx abdA)/Df(Ubx abdA AbdB)* transheterozyogote (R), Cut positive ASPs are detected in metameres 1–9. And frequent DT fusion occurs only in DT9/10. The airway specific overexpression of *Ubx* (S) or *abdA* (T) partially restores DT fusion (arrows). Note that *bnl* expression guiding DT branches are intact in the absence of BX-C complex [130].
Scales bars: 50um.
(TIF)

S4 Fig *salm* dependent and independent airway tube tapering by BX-C and a potential *salm* target for tube tapering. (A-C, F) DT tube tapering visualized by Gasp and PTP10D.
Compared to the control (A) where DT3 is slightly thicker than DT2, in *bxd^100^* (B) or *bxd^113^* (C) mutants, DT3 becomes comparable or slightly narrower than DT2. In BX-C triple mutants (F), the posterior metameres tend to show slightly thicker DT tubes (arrows).
(D, E) Ubx expression in BX-C mutants a stage 13/14.
In *bxd^113^* mutants (D), Ubx expression in DT3 becomes comparable to the level in DT2 while in *abdA AbdB* double mutants, Ubx expression in DT4-10 becomes comparable to the level in DT3.
(G-J) Effects of *abdA* and/or *AbdB* mutations on Salm expression in DT.
In the control (G), Salm expression level forms a posterior-to-anterior gradient. In *abdA* mutants (H), Salm expression in the central metameres becomes comparable to the more anterior metameres while in *AbdB* mutants (I) Salm expression in the posterior metameres becomes comparable to the central metameres. In *abdA AbdB* double mutants (J), the Salm gradient becomes more flat, but a weak gradient is still detected.
(K-M) Effects of *salm* on Tnc expression in DT.
In the control (K) at stage 16, Tnc is secreted into the lumen but is locally detected in DT and TC1, both of which express *salm*. Expression of both Tnc and Salm appears slightly stronger in fusion points. Note that Tnc is also expressed in hindgut and the proximal part of PSP. Upon overexpression of *salm* (L) Tnc is detected in lumens of additional branches like TC (arrow). In Df(*salm/salr*) mutants where both *salm* and the neighboring *spalt-related* (*salr*) are deleted, Tnc expression in the airway is lost (M, asterisk), although its expression in PSP and hindgut remains.
(N) In *Df(salm/salr)* mutants, TC branches in the posterior metameres are thicker than in the anterior.
Scales bars: 50um.
(TIF)

S1 Table DT thickness of various tube diameter mutants. Genotypes are; Wild type = Canton S, *bxd* = *bxd^113^*, *abdA* = *abdA^MX^*, *AbdB* = *AbdB^M1^* / *AbdB^M5^*, *abdA AbdB* = *abdA^D24^ AbdB^D18^*.
Table A. Raw data of DT thickness in micrometers. For each genotype, 3 embryos were measured for DT thickness. 3 points of each DT near the base of DB were measured.
Table B. Statistics of the raw data. Average thickness, standard deviation (SD) and p-value of student t-test are shown for each DT. For p-value, comparison between WT and each mutant is shown. For wt, bxd, abdA, AbdB single or abdA AbdB double mutants, additional comparison is shown between different metameres or between mutants. p<0.05 is judged to be significant.
(XLSX)
