# Supplementary figures and images for: The Intersection of the Extrinsic Hedgehog and WNT/Wingless Signals with the Intrinsic Hox Code Underpins Branching Pattern and Tube Shape Diversity in the Drosophila Airways
Source: PLoS Genet. 2015 Jan 23;11(1):e1004929. doi: 10.1371/journal.pgen.1004929 (PMC4304712; doi:10.1371/journal.pgen.1004929)

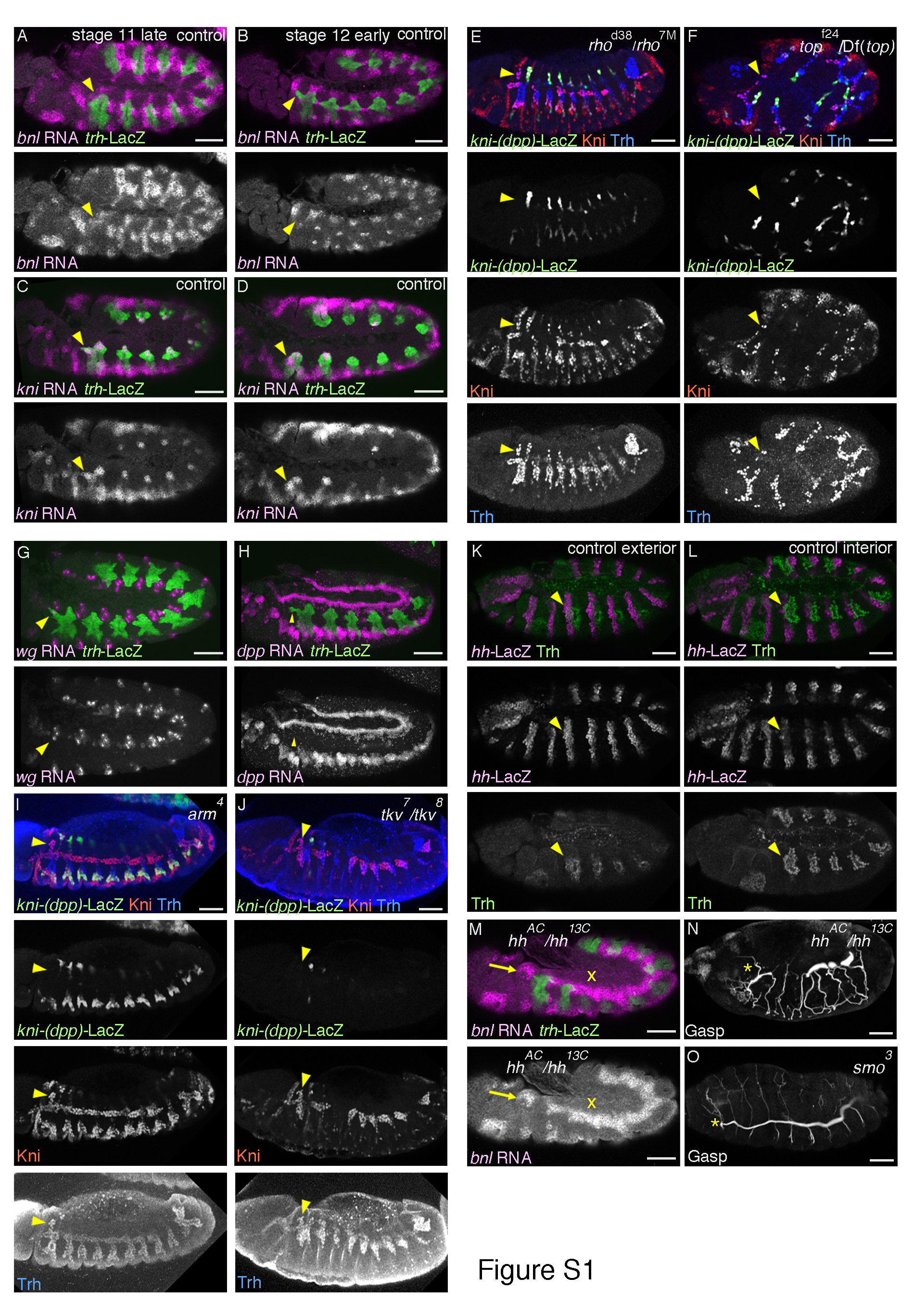

Supplement: S1 Fig — DTa1 is marked with arrowhead or asterisks in the different panels. (A-D) bnl signaling. (A, B) Expression of bnl. (A) At late stage 11, corresponding to the 6 primary branches of the airway (marked with trh-lacZ), 6 patches of surrounding cells express bnl for Tr1. (B) At stage 12, DTa1 appears to extend toward bnl expression corresponding to the DB0 position. (C, D) Expression of kni RNA. Compared to the control (C), in btl mutants (D) at early stage 12, kni induction in DB is variably abolished while its DTa1 expression is comparable. Different magnification was used to image the same embryo in Fig. 1B and S1C Fig. (E-F) dEGFR signaling. In either rho mutant (E) or dEGFR mutant (F), Kni expression in DTa1/CB at stages 13–14 is comparable to the control (Fig. 1D). (G-J) wg and dpp signaling. wg RNA distribution (G) relative to DT or dpp RNA expression (H) in the dorsal and the lateral ectodermal stripes at stages11/12 are comparable in all metameres. (I) In arm mutants at stage 13, expression of Kni as well as kni-(dpp)-LacZ in DB/LT/GB are comparable to the control (Fig. 1D). (J) In tkv mutants at stage 13, kni-(dpp)-LacZ expression in DB/LT/GB is variably abolished while Kni expression in DTa1/CB is comparable to the control (Fig. 1D). (K-O) hh signaling. (K, L) Exterior (K) or interior (L) sections of Hh expression monitored with a hh enhancer trap line. hh is detected just above the anterior part of the invaginated airway primodia for all metameres (marked with Trh). (M) In hh mutants at stage 12, bnl expression corresponding to DB extension is significantly decreased (cross). The ectodermal patches of bnl expression corresponding to DT extension become continuous. (N, O) Gasp positive airway branching in hh or smo mutants. (N) hh mutants have variable defects including loss or stalling of branches (DB, DT, VB or CB) or loss of metameres. CB does not form dorsal extension. (O) smo mutants often show CB misrouting phenotypes. Scales bars: 50um. (TIF) [file pgen.1004929.s001.tif]

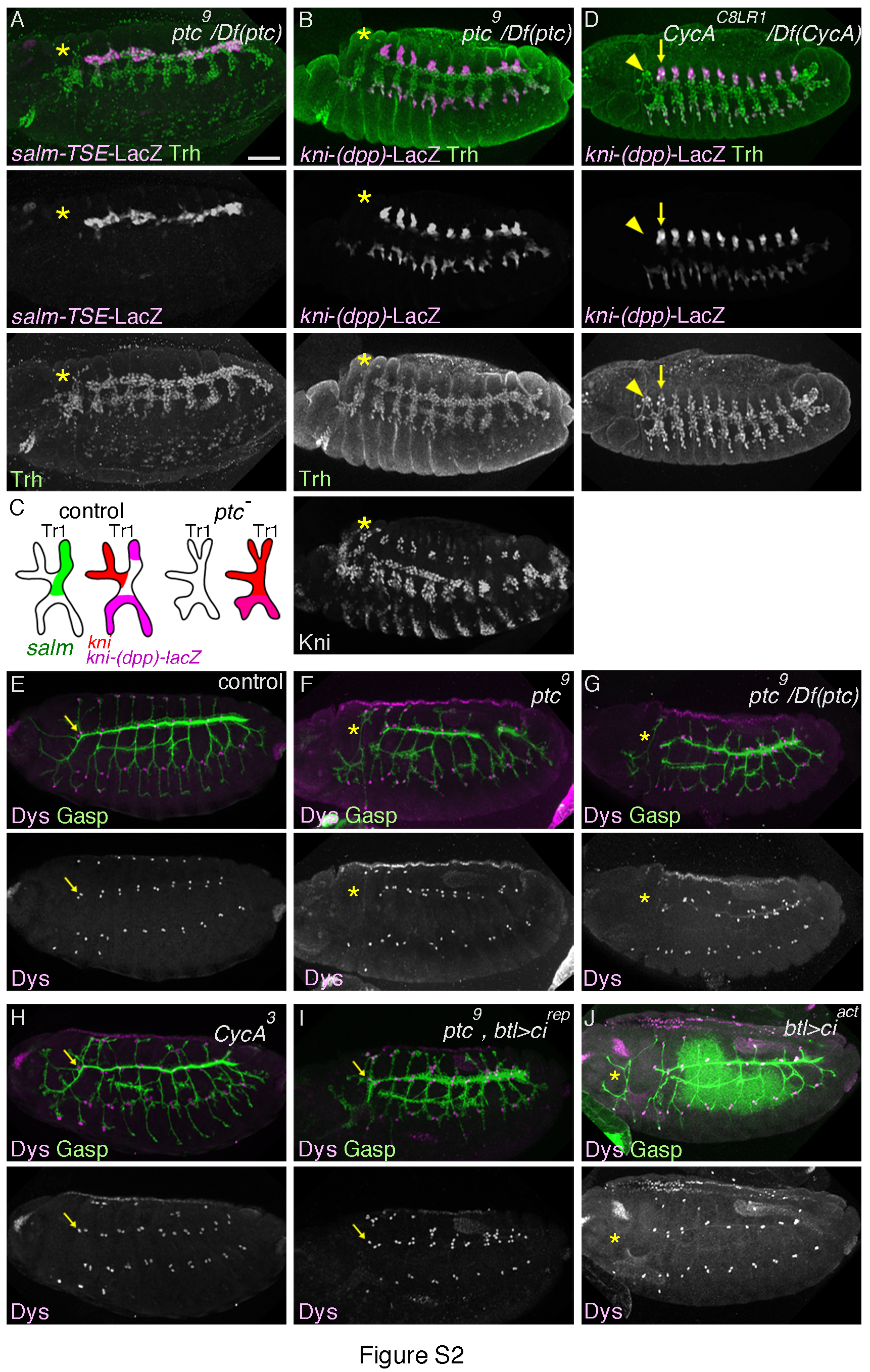

Supplement: S2 Fig — (A-D) Effects of ptc or CycA mutations on expression of kni or salm. A summary of ptc mutant phenotypes is shown in (C). (A, B) In ptc/Df(ptc) mutants, expression of salm-TSE-lacZ (A) and kni-(dpp)-lacZ (B) is lost in the dorsal part of the metamere 1 (asterisks) while Kni protein is expressed in the whole distal trachea in Tr1 (B, bottom panel). (D) In CycA mutants, where the cell number in the airways is reduced nearly to half [38], DBs in all metameres express kni-(dpp)-lacZ comparably (arrows) and CB formation can occur (arrowheads). (E-J) Stage 14–15 embryos stained with Gasp and Dys. In the control (E), the fusion point of DT1/2 is occupied by two fusion cells originating from DTp1 and DTa2 (arrow). In ptc mutants (F, G), dys expression is not detected in the positions of DT1p (and LT1p) (asterisks) while CycA mutant embryos (H) are comparable to the control (arrow). btl-Gal4 driven Cirep (I) restores Dys positive fusion cells in DTp1 as well as fusion of DT1/2 (arrow). Overexpression of Ciact with btl-Gal4 (J) can mimic the phenotypes of ptc mutants. Dys expression in DTp1 position is lost and is accompanied by fusion defects of DT1/2 (asterisk). Scales bars: 50um. (TIF) [file pgen.1004929.s002.tif]

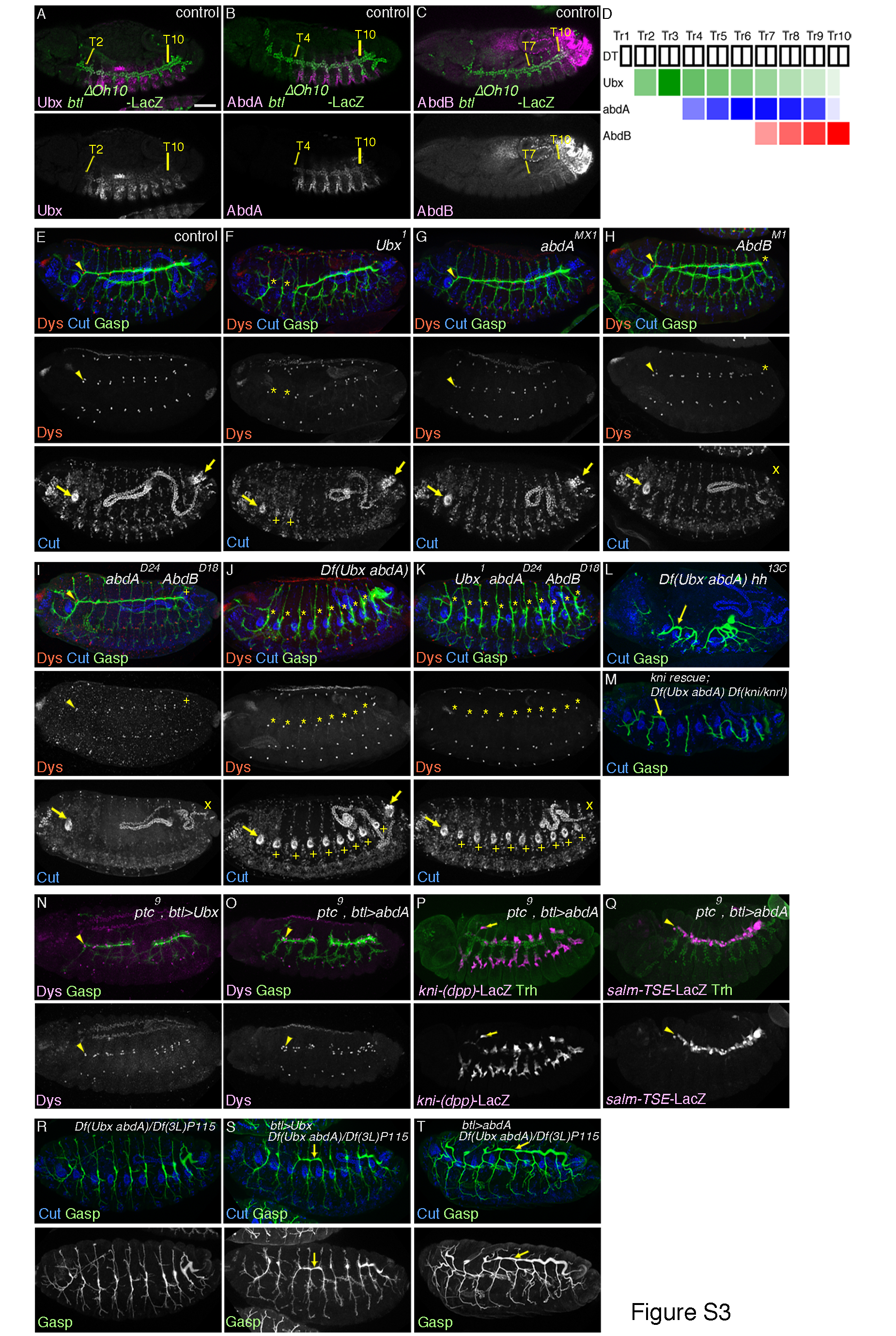

Supplement: S3 Fig — (A-D) Expression of BX-C genes at stage 13 visualized by the respective antibodies. A summary of the expression patterns is shown in (D). (A) Ubx expression starts from metamere 2 and peaks at metamere 3. From metamere 3 to 10, Ubx expression forms a gradient. (B) abdA expression starts from metamere 4 and peaks at metamere 6. From metamere 7 to 10, abdA expression forms a gradient. Only the anterior half of metamere 10 expresses Ubx and abdA. (C) AbdB expression starts in metamere 7 and peaks at metamere 10, forming a single gradient. (E-K) Airway branching phenotypes of BX-C mutants assessed at stage 15 with expression of Gasp, Dys and Cut. In the control (E), DT is continuous with a pair of Dys positive fusion cells at each fusion point (arrowhead for DT1/2). The terminal parts of the airway are plugged with Cut positive ASP and PSP (arrows) [125]. In Ubx mutants (E), metameres 2/3 are transformed to metamere 1 as judged by the loss of fusion cells at the position of DTa (asterisks) and gain of Cut positive ASP (marked with +). While abdA mutants (F) are largely normal, both AbdB single mutants (H) and abdA AbdB double mutants (I) have an ectopic fusion cell in DTp10 (asterisks), in addition to loss of Cut positive PSP (crosses). In Df(Ubx abdA) mutants (J), in addition to metameres 2/3, metameres 4–6 are transformed to Tr1 while transformation is variable in Tr7-9. Namely, Dys-positive fusion cells at the position of DTa are lost in Tr2-8 while Tr 9 has 1–2 fusion cell. Fusion of DT9/10 is frequently observed. Cut positive ectopic ASPs are detected in Tr2-9 (marked by +). DTp diameter in Tr7-10 looks thicker compared to the more anterior metameres. In Ubx abdA AbdB triple mutants (K), all metameres are transformed to Tr1. Cut-positive PSP is lost (cross). Instead, Cut positive ASP (+) as well as loss of fusion cell fates at the position of DTa (asterisks) is observed in all metameres. Note that in Ubx, Df(Ubx abdA) and Ubx abdA AbdB mutants, CB-like branches are [file pgen.1004929.s003.tif]

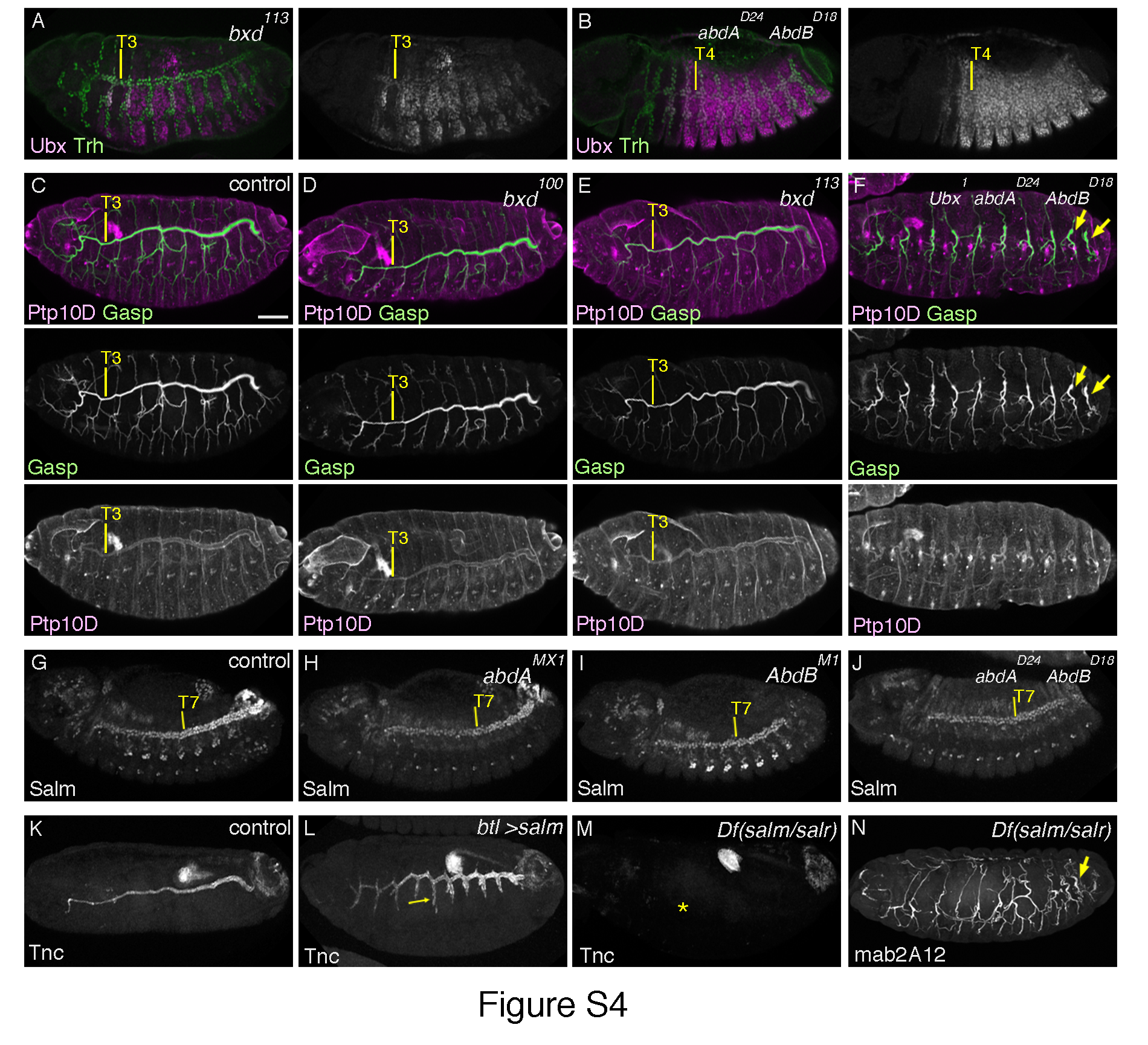

Supplement: S4 Fig — (A-C, F) DT tube tapering visualized by Gasp and PTP10D. Compared to the control (A) where DT3 is slightly thicker than DT2, in bxd100 (B) or bxd113 (C) mutants, DT3 becomes comparable or slightly narrower than DT2. In BX-C triple mutants (F), the posterior metameres tend to show slightly thicker DT tubes (arrows). (D, E) Ubx expression in BX-C mutants a stage 13/14. In bxd113 mutants (D), Ubx expression in DT3 becomes comparable to the level in DT2 while in abdA AbdB double mutants, Ubx expression in DT4-10 becomes comparable to the level in DT3. (G-J) Effects of abdA and/or AbdB mutations on Salm expression in DT. In the control (G), Salm expression level forms a posterior-to-anterior gradient. In abdA mutants (H), Salm expression in the central metameres becomes comparable to the more anterior metameres while in AbdB mutants (I) Salm expression in the posterior metameres becomes comparable to the central metameres. In abdA AbdB double mutants (J), the Salm gradient becomes more flat, but a weak gradient is still detected. (K-M) Effects of salm on Tnc expression in DT. In the control (K) at stage 16, Tnc is secreted into the lumen but is locally detected in DT and TC1, both of which express salm. Expression of both Tnc and Salm appears slightly stronger in fusion points. Note that Tnc is also expressed in hindgut and the proximal part of PSP. Upon overexpression of salm (L) Tnc is detected in lumens of additional branches like TC (arrow). In Df(salm/salr) mutants where both salm and the neighboring spalt-related (salr) are deleted, Tnc expression in the airway is lost (M, asterisk), although its expression in PSP and hindgut remains. (N) In Df(salm/salr) mutants, TC branches in the posterior metameres are thicker than in the anterior. Scales bars: 50um. (TIF) [file pgen.1004929.s004.tif]
